# Cytokine-overexpressing dendritic cells for cancer immunotherapy

**DOI:** 10.1038/s12276-024-01353-5

**Published:** 2024-12-02

**Authors:** Joonsu Han, Hua Wang

**Affiliations:** 1 Department of Materials Science and Engineering, University of Illinois at Urbana-Champaign, Urbana, IL USA; 2 Cancer Center at Illinois (CCIL), Urbana, IL USA; 3 Department of Bioengineering, University of Illinois at Urbana-Champaign, Urbana, IL USA; 4 Carle College of Medicine, University of Illinois at Urbana-Champaign, Urbana, IL USA; 5 Beckman Institute for Advanced Science and Technology, University of Illinois at Urbana-Champaign, Urbana, IL USA; 6 Materials Research Laboratory, University of Illinois at Urbana-Champaign, Urbana, IL USA; 7 Institute for Genomic Biology, University of Illinois at Urbana-Champaign, Urbana, IL USA

**Keywords:** Cytokines, Tumour immunology, Cancer immunotherapy

## Abstract

Dendritic cells (DCs), the main type of antigen-presenting cells in the body, act as key mediators of adaptive immunity by sampling antigens from diseased cells for the subsequent priming of antigen-specific T and B cells. While DCs can secrete a diverse array of cytokines that profoundly shape the immune milieu, exogenous cytokines are often needed to maintain the survival, proliferation, and differentiation of DCs, T cells, and B cells. However, conventional cytokine therapies for cancer treatment are limited by their low therapeutic benefit and severe side effects. The overexpression of cytokines in DCs, followed by paracrine release or membrane display, has emerged as a viable approach for controlling the exposure of cytokines to interacting DCs and T/B cells. This approach can potentially reduce the necessary dose of cytokines and associated side effects to achieve comparable or enhanced antitumor efficacy. Various strategies have been developed to enable the overexpression or chemical conjugation of cytokines on DCs for the subsequent modulation of DC–T/B-cell interactions. This review provides a brief overview of strategies that enable the overexpression of cytokines in or on DCs via genetic engineering or chemical modification methods and discusses the promise of cytokine-overexpressing DCs for the development of new-generation cancer immunotherapy.

## Enhancing Dendritic Cells for Improved Cancer Immunotherapy

Cancer immunotherapy has transformed cancer treatment, particularly with immune checkpoint blockades and CAR T cell therapy. However, cancer vaccines have been less effective. Researchers investigated ways to improve cancer vaccines by targeting dendritic cells and cytokines. DCs are immune cells that present antigens to T and B cells, starting immune responses. The study involved genetically or chemically modifying DCs to produce more cytokines like IL-2 and IL-12. This aimed to better activate T cells and enhance the immune response against tumors. Results showed that these modified DCs significantly increased T cell responses and reduced tumor growth in mice. Researchers concluded this could improve cancer vaccine effectiveness and reduce side effects. Future studies may refine cytokine combinations and delivery methods for clinical use.

This summary was initially drafted using artificial intelligence, then revised and fact-checked by the author.

## Introduction

Cancer immunotherapy has shifted the paradigm for cancer treatment over the past few decades, especially with the success of immune checkpoint blockade and chimeric antigen receptor (CAR) T-cell therapy^1^. Cancer vaccines that aim to modulate antigen-presenting cells (e.g., dendritic cells (DCs)) with tumor antigens and adjuvants, with the goal of eliciting potent and persistent tumor-specific cytotoxic T lymphocyte (CTL) responses, have also achieved significant progress^2,3^. DCs are the prominent type of antigen-presenting cells in the body and serve as conduits for communication within the immune system, as reflected by their roles as sentinels to capture and process antigens from the surrounding environment. Upon encountering antigens, DCs process and present the antigens and migrate to lymphatic tissues, where they present the antigens to T and B cells, thereby driving the clonal expansion of antigen-specific T and B cells for the orchestration of adaptive immune responses^4^. DCs should be capable of efficiently presenting antigens via major histocompatibility complexes (MHCs), expressing sufficient costimulatory signals on the cell membrane, exhibiting a phenotype with superior migratory and tissue penetration properties, and releasing immunomodulatory molecules to further modulate the proliferation and function of T and B cells and enable the optimal priming of antigen-specific T and B cells^5^. However, DCs are often heterogeneous and have multifaceted functions in immunomodulation by priming and regulating the activation, differentiation, and function of tumor antigen-specific CTLs, CD4^+^ T helper cells, regulatory T cells, and B cells^6,7^. In addition to tumor antigens and adjuvants, additional immunomodulatory signals are often needed to control the T- and B-cell priming functions of DCs^8,9^.

**Fig. 1:**
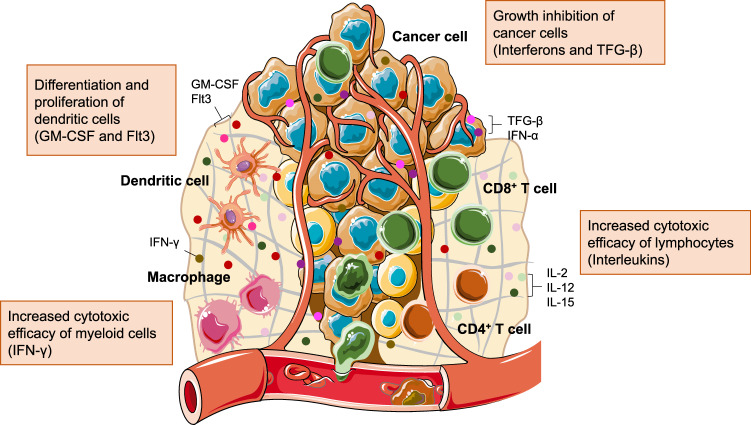
Functions of proinflammatory cytokines in the tumor microenvironment. Schematic illustration of the major immune cells and representative proinflammatory cytokines found in the tumor microenvironment. Proinflammatory cytokines can regulate tumor growth, the proliferation of dendritic cells, and the cytotoxic activities of lymphocytes and myeloid cells to increase antitumor efficacy.

Cytokines play critical roles in maintaining the survival and proliferation of immune cells, controlling the differentiation process and phenotypes of immune cells, and orchestrating interactions between different types of immune cells^10–12^. For example, IL-2, IL-12, and IL-15 are able to drive the proliferation of effector CD8^+^ and CD4^+^ T cells in peripheral lymphoid tissues and the tumor microenvironment, thus amplifying the overall CTL response and antitumor efficacy^13,14^. Granulocyte‒macrophage colony-stimulating factor (GM-CSF) can induce the differentiation and proliferation of DCs^15^. Certain cytokines, such as interferons and TGF-β, can also directly affect the survival and growth of tumor cells (Fig. 1)^16–18^. In clinical settings, cytokine therapy has been actively evaluated for treating various types of cancer. To date, IL-2 has been approved by the FDA as a treatment for advanced renal cell carcinoma and metastatic melanoma, and IFN-α has been approved as a treatment for hairy cell leukemia, follicular non-Hodgkin lymphoma, melanoma and AIDS-related Kaposi’s sarcoma^19–21^. However, the short half-life, low patient response rate, and severe side effects of proinflammatory cytokines have limited their utility for cancer treatment^22^. In particular, the systemic administration of cytokines often causes system-level inflammation and off-target side effects, resulting in the failure of most cytokine therapies in clinical trials^23^. These issues have motivated the development of new strategies that enable the controllable exposure of cytokines to immune cells of interest. For example, the controlled expression of cytokines in DCs holds great promise for precisely orchestrating T- and B-cell priming processes, potentially achieving increased antitumor efficacy with reduced side effects^24^.

Upregulating the expression of cytokines in DCs is promising for finely tuning adaptive immune responses and the overall therapeutic efficacy of immunotherapies against malignant cancers^24^. Adjuvants or other molecules that can bind to Toll-like receptors (TLRs) on the membrane of DCs can often stimulate the intracellular pathways associated with cytokine expression and have been widely incorporated into the design of vaccines and other immunotherapies. These efforts have been reviewed by others^25–27^ and will not be the focus of this review. Genetic engineering of DCs with cytokine-encoding plasmids has been actively explored to directly control the expression levels of specific cytokines. The engineered DCs overexpress specific cytokines either intracellularly or on the cell membrane. For the former, cytokines either drive the activation and proliferation of DCs themselves or are gradually released to modulate the extracellular environment^28^. For the latter, cytokines are displayed on the surface of DCs to orchestrate intercellular interactions with other immune cells, such as CD8^+^ T cells^29,30^. In addition to genetic engineering methods, physical adsorption or chemical conjugation of cytokines to the membrane of DCs has also been attempted to provide a continuous activation signal to the interacting cells (e.g., T cells)^31^. This review provides a brief overview of strategies that have been utilized to upregulate the expression of cytokines in DCs or surface display of cytokines on DC membranes and their applications for the development of improved cancer immunotherapies. We discuss the current research progress in preclinical and clinical settings, potential challenges for each strategy, and future directions for the development of cytokine expression or display approaches.

## Cytokine therapy in the clinic

Over the past few decades, various types of proinflammatory cytokines have been evaluated for cancer treatment in clinical trials, many of which have shown CTL responses and antitumor efficacy against blood and solid tumors at different stages of cancer (Table 1). Among them, IFN-α and IL-2 have been approved by the FDA, and several other proinflammatory cytokines are actively being explored in numerous ongoing clinical trials^32^. In 1986, IFN-α became the first FDA-approved cytokine to treat human cancer, particularly hairy cell leukemia^33^. Since then, IFN-α has been extensively tested as a treatment for various types of hematological malignancies and solid tumors^34^. To date, IFN-α has obtained FDA approval as a primary treatment for individuals with metastatic renal cell carcinoma, AIDS-related Kaposi’s sarcoma, follicular lymphoma, chronic myelogenous leukemia, hairy cell leukemia, and cervical intraperitoneal neoplasms and as an adjuvant therapy for patients with completely resected stage III or IV melanoma^22,23^. Nevertheless, the utilization of IFN-α as an anticancer therapy is not without challenges, as it exhibits significant dose-dependent toxicity and often causes symptoms, including fever, fatigue, headache, and gastrointestinal disturbances^35^. Additionally, neuropsychiatric manifestations, such as depression, confusion, and mania, coupled with electroencephalographic alterations, have been documented, and instances of suicide have been associated with IFN-α use^35^.

**Table 1:** Representative cytokine therapies investigated in clinical trials.

Type of cytokine	Trial ID	Phase	Condition	Drug	Expected completion
IL-2	NCT02086721	1	Solid Tumors	L19-IL2	2017-05
	NCT05307874	1, 2	Solid Tumors	Proleukin Injectable Product	2024-12
	NCT00200577	3	Melanoma	TIL + IL2	2012-03
	NCT01416831	2	Metastatic Melanoma	High-dose IL-2	2024-06
	NCT00100906	2	Kidney Cancer	IL-2, ATRA	2013-07
	NCT00539695	2	Acute Lymphoblastic Leukemia	IL-2	2014-03
	NCT00554515	2	Metastatic Renal Cell Carcinoma	High-dose IL2	2013-10
	NCT05336409	1	R/R CD19-Positive B-Cell Malignancies, Indolent Non-Hodgkin Lymphoma	CNTY-101, IL-2, Lymphodepleting Chemotherapy	2027-08
	NCT02919644	2	Stage IV Colorectal Cancer	Autologous dendritic cells, IL2	2024-04
	NCT04155710	1, 2	Chronic Lymphocytic Leukemia, Small Lymphocytic Lymphoma	IOV-2001, IL-2	2024-07
	NCT01995344	2	Metastatic Melanoma	Cyclophosphamide, Fludarabine, TIL, IL-2	2015-07
	NCT00058786	1	Chronic Lymphocytic B-Leukemia	IL-2-secreting CD40L-expressing autologous B-CLL	2010-03
IFN-α	NCT02199327	4	Conjunctival/corneal Intraepithelial Neoplasia	Mitomycin C, IFN-α	2017-07
	NCT00002621	2	Leukemia Lymphoma	Recombinant IFN-α	2005-09
	NCT00082719	1	Bladder Cancer, Urethral Cancer	Recombinant IFN-α	2015-01
	NCT02838342	2	Neuroendocrine Tumors	Metronomic cyclophosphamide, IFN-α	2021-06
	NCT00002965	2	Brain and CNS Tumors	Recombinant IFN-α	2003-03
	NCT00276536	1	Breast Cancer, Kidney Cancer, Lymphoma, Melanoma, Multiple Myeloma Sarcoma	IFN-α	2004-01
	NCT00003542	1, 2	Kidney Cancer	Pegylated IFN-α	2004-04
	NCT00002506	2	Cervical Cancer, Esophageal Cancer, Head and Neck Cancer, Lung Cancer	Recombinant IFN-α, Isotretinoin	1999-02
	NCT00053820	3	Kidney Cancer	Aldesleukin, IFN-α, Fluorouracil	2006-12
	NCT04116502	3	Polycythemia Vera	Ruxolitinib, Hydroxycarbamide, IFN-α	2028-02
	NCT02737046	2	Adult T-cell Leukemia–Lymphoma ATLL	Belinostat, Zidovudine, IFN-α	2025-12
	NCT00003656	2	Kidney Cancer	IFN-α, Tretinoin liposomes	2008-05
IL-12	NCT01417546	1	Epithelial Neoplasms, Malignant Epithelial/Mesenchymal Tumors	NHS-IL-12	2021-10
	NCT04471987	1	Advanced Solid Tumors	IL12-L19	2023-12
	NCT02483312	1	Acute Myeloid Leukemia	IL-12	2024-08
	NCT00005604	1	Adult Solid Tumors	IL-12, Aldesleukin	2003-07
	NCT00015977	2	Prostate Cancer	PSA prostate cancer vaccine, IL-12	2005-01
	NCT00004074	1	Advanced Adult Primary Liver Cancer, Anaplastic Thyroid Cancer, Bone Metastases	IL-12, ABI-007/Carboplatin/Trastuzumab	2009-02
	NCT00323206	1	Malignant Melanoma	IL-12p DNA	2008-04
	NCT00004893	2	Breast Cancer	IL-12	2002-02
	NCT00016289	2	Primary Peritoneal Cavity Cancer, Recurrent Ovarian Epithelial Cancer	IL-12	2007-07
	NCT05756907	1, 2	Advanced Solid Tumors, Platinum-resistant Ovarian Cancer	SON-1010	2025-05
	NCT00028535	1	Male Breast Cancer, Recurrent Breast Cancer, Recurrent Endometrial Carcinoma, Recurrent Gastric Cancer	Trastuzumab, Paclitaxel, IL-12	2009-02
	NCT00003210	2	B-cell Lymphoma, Recurrent Adult Diffuse Large/Mixed Cell Lymphoma	IL-12	2003-11
IL-15	NCT01021059	1	Melanoma, Carcinoma, Renal Cell	rh IL-15	2016-10
	NCT01572493	1	Lymphoma, Carcinoma	rh IL-15	2019-07
	NCT02689453	1	T-cell Lymphoma, T-cell Leukemia	IL-15, Alemtuzumab	2021-06
	NCT03759184	1	Leukemia, Lymphocytic Chronic B-Cell	rhIL-15, Obinutuzumab	2021-10
	NCT03388632	1	Metastatic Solid Tumors, Treatment-refractory Cancers	rhIL-15, Ipilimumab, Nivolumab	2024-12
	NCT04185220	1	Adult T-cell Lymphoma/Leukemia	rhIL-15, Mogamulizumab	2022-05
	NCT03905135	1	Peripheral T-cell Lymphoma, Anaplastic Large Cell Lymphoma	rhIL-15, Avelumab	2022-05
	NCT01727076	1	Head and Neck Squamous Cell Carcinoma, Recurrent Non-Small Cell Lung Carcinoma, Recurrent Renal Cell Carcinoma	rhIL-15	2016-06

IL-2 received FDA approval for treating metastatic renal cell carcinoma in 1992 and metastatic melanoma in 1993^36^. IL-2 plays an important role in regulating both the adaptive and innate immune systems. By functioning as a T-cell-stimulating agent during the initiation of immune responses, IL-2 can facilitate the activation and proliferation of antigen-specific CD8^+^ T cells, effector CD4^+^ T cells, and natural killer (NK) cells^37^. Over the past few decades, IL-2 treatment has been scrutinized across various dose ranges, schedules, and administration routes in the pursuit of maximizing efficacy while minimizing toxicity^38^. Due to the short blood half-life and poor pharmacokinetics of IL-2 and the imperative to achieve a potent immunomodulatory effect, a high dose of IL-2 is often needed, inevitably causing severe systemic toxicity, including vascular leak syndrome, pulmonary edema, hypotension, acute renal insufficiency, and, rarely, myocarditis^39^.

Other interleukin family cytokines have also been actively explored in clinical trials. IL-15 was shown to improve the proliferation and cytotoxic function of CD8^+^ T cells and NK cells and thus lead to an enhanced CTL response and therapeutic efficacy^40^. Despite its therapeutic potential, over 170 global clinical trials involving IL-15 for cancer treatment have been frequently limited by adverse effects, including bleeding, papilledema, uveitis, pneumonitis, duodenal erosions, and fatalities^41^. Similarly, clinical trials involving IL-7, IL-12, and IL-21 have documented side effects such as fatigue, dyspnea, acidosis, leukopenia, elevated liver function, and even death^42–44^. While these cytokine therapies show promise in eliciting a robust CTL response against different types of blood and solid tumors, the associated adverse effects necessitate the development of strategies for the controlled exposure of proinflammatory cytokines to immune cells.

## Intrinsic cytokines in DCs

**Fig. 2:**
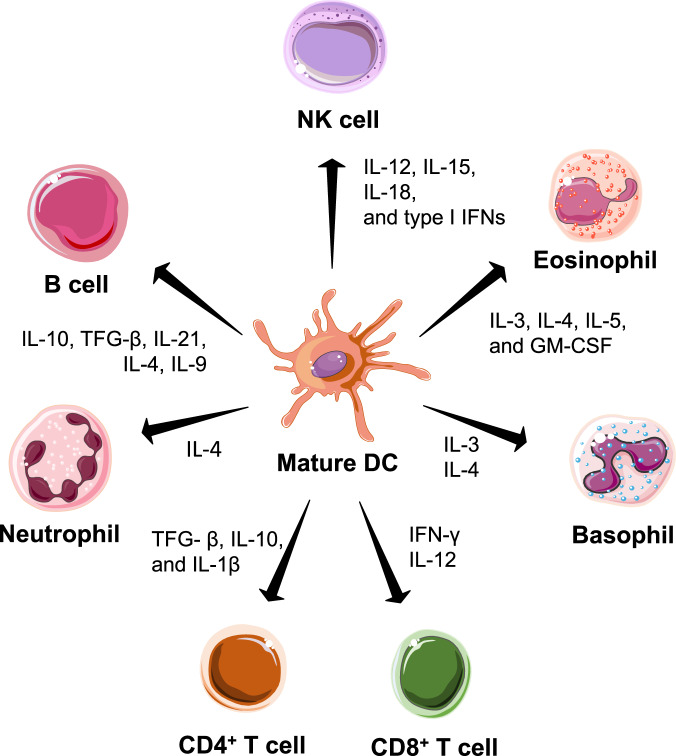
Dendritic cells express various types of cytokines to modulate interacting or surrounding immune cells. DCs express a wide spectrum of interleukins (IL-12, IL-15, IL-18, IL-10, IL-21, IL-4, IL-9, IL-1β, IL-3, and IL-5), type I interferons, transforming growth factor β, and chemokines (GM-CSF) to stimulate or suppress interacting or surrounding T cells, B cells, NK cells, neutrophils, eosinophils, and basophils.

DCs can intrinsically express various types of cytokines that either drive self-proliferation and differentiation or modulate interacting lymphocytes and other immune cells^45^. For example, DCs express GM-CSF, which can maintain the survival and proliferation of DCs and eosinophils^46^. During the CD8^+^ T-cell priming process, DCs express and release IFN-γ and IL-12 to promote the activation and maturation of CD8^+^ T cells, which is critical for generating robust CTL responses and antitumor efficacy. DCs can also express transforming growth factor-β (TGF-β), IL-10, and IL-1β to regulate the differentiation of CD4^+^ T cells and modulate T helper 1 and 2 responses^47–49^. In addition to T cells, DCs also express and release various types of cytokines, including IL-10, TGF-β, IL-21, IL-4, and IL-9, to modulate the differentiation and function of B cells and orchestrate antigen-specific humoral responses^50^. DCs also play a crucial role in mediating innate immune responses. One notable example is the ability of DCs to modulate the function of NK cells via the release of cytokines such as IL-12, IL-15, IL-18, and type I interferons^51^. DCs can also express and release IL-3 and IL-4 to regulate the recruitment and differentiation of neutrophils and basophils and IL-3, IL-4, IL-5, and GM-CSF to modulate eosinophils (Fig. 2)^52,53^. However, the level of cytokines intrinsically expressed and released by DCs is often low, depending on the status of inflammation and the type of tissue microenvironment^54–56^. Additionally, the mix of proinflammatory and anti-inflammatory cytokines released by DCs makes a predicting the overall immunomodulatory effects difficult^57^. These issues have motivated the development of new strategies to upregulate the expression of specific cytokines in DCs and improve the precise priming of antigen-specific T and B cells and other immune cells.

## Overexpression of soluble cytokines in DCs

**Fig. 3:**
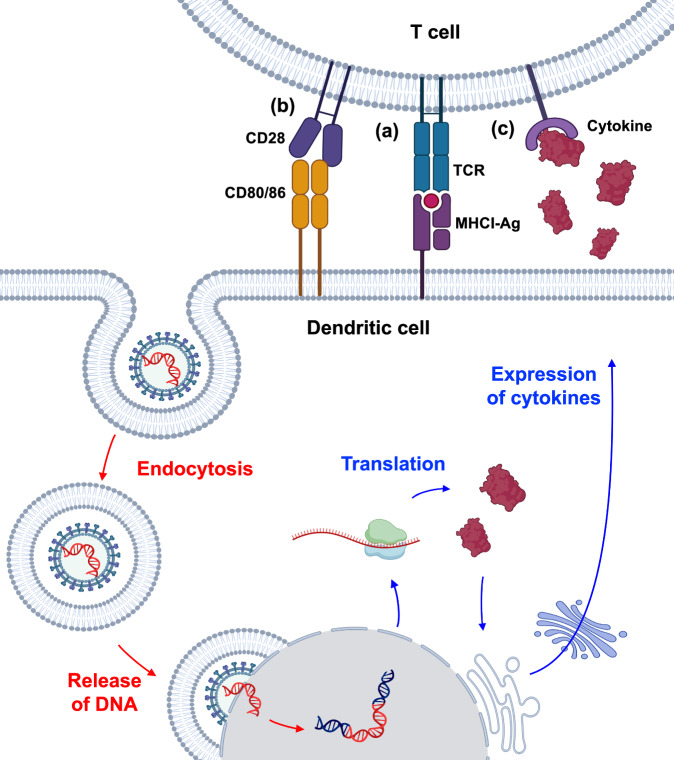
The overexpression of cytokines in DCs via viral transduction orchestrates T-cell priming processes. Cytokine-encoding DNA is delivered to DCs via viral vectors and becomes integrated into the genome of host DCs for subsequent transcription and translation into cytokines. The cytokines expressed by DCs could facilitate the priming process of T cells. During T-cell priming, (**a**) stimulatory signals (MHCI-Ag-TCR), (**b**) costimulatory signals (CD80-CD28), and (**c**) DC-released cytokines collectively contribute to the expansion of antigen-specific T cells.

DC-mediated priming of antigen-specific T cells is a crucial step in adaptive immunity. For a desirable priming process, T cells need three signals: MHC–antigen complexes presented by DCs, costimulatory signals from the DC membrane, and soluble cytokines (e.g., IL-2) that can maintain the survival and proliferation of T cells^58^. These processes often require the presence of DCs with the right phenotype, proper activation status, and high antigen presentation efficiency, as well as the exposure of a sufficient amount of cytokines to T cells. The incorporation of an adjuvant that can activate DCs and facilitate antigen delivery into DCs are common strategies to improve the activation status and antigen presentation efficiency of DCs, but additional cytokines are often needed to maintain the survival and proliferation of DCs and T cells^59^. For example, exogenous GM-CSF can be added to facilitate the proliferation and maturation of DCs^60^. Exogenous IL-2 and IL-15 are also commonly used to increase the expansion of T cells^61^. However, while the simple addition of soluble cytokines to DC–T-cell cocultures may improve the expansion of DCs or T cells, the seamless integration of cytokine-mediated cell modulation and DC-mediated T-cell priming processes remains a significant challenge. To this end, the overexpression of cytokines in DCs via genetic methods has been actively pursued as a means to better orchestrate DC-mediated T-cell priming processes (Fig. 3)^62^. For example, GM-CSF-overexpressing DCs could exhibit enhanced survival, persistence, and activation for the improved priming and expansion of T cells^63,64^. IL-2-overexpressing DCs can simultaneously prime antigen-specific T cells and gradually release IL-2 to drive the expansion of primed T cells^65^.

### Overexpression of soluble DC-effector cytokines

**Fig. 4:**
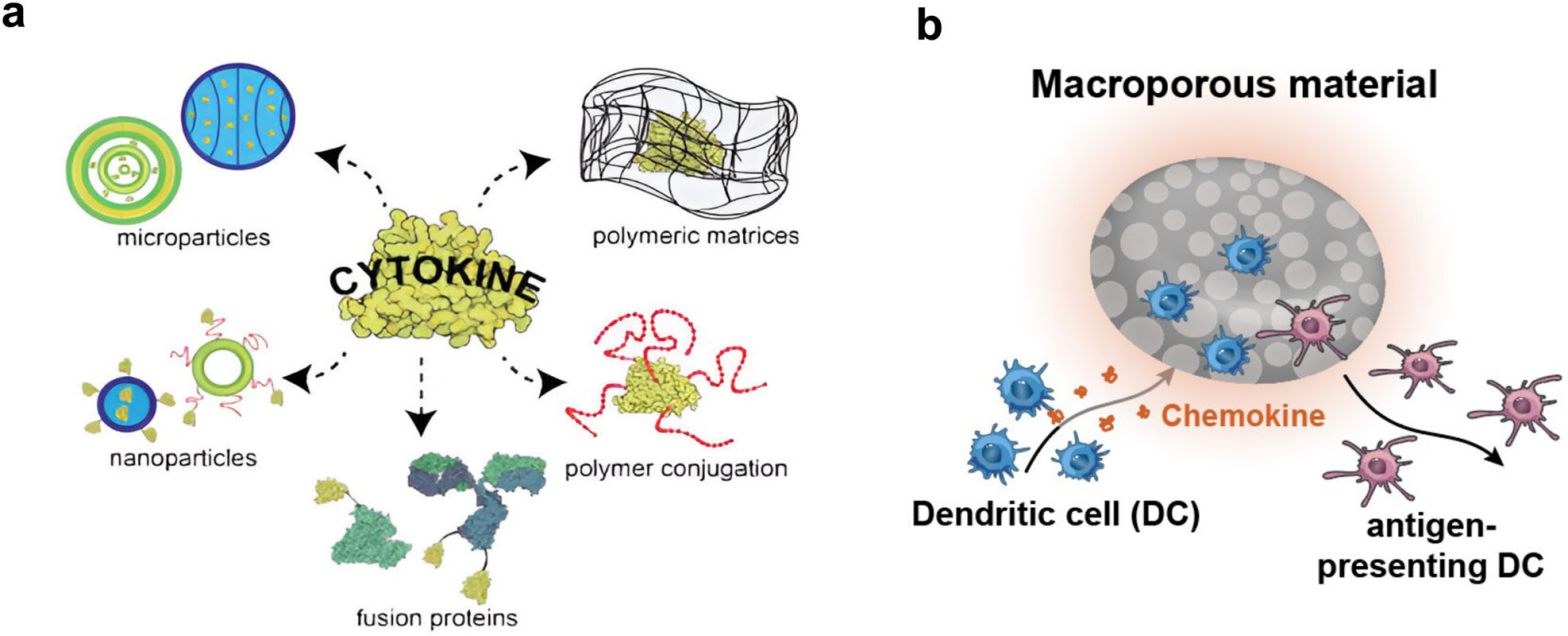
Material-based approaches to control the exposure of DCs to cytokines. **a** Schematic illustration of various material approaches used to deliver immunomodulatory cytokines. Adapted from Pires et al.^68^. **b** Macroporous hydrogels loaded with chemokines (e.g., GM-CSF) for in situ DC recruitment and modulation. Adapted from Wang et al.^70^.

The ability of DCs to present antigens, express costimulatory signals, and prime T and B cells is intrinsically heterogeneous. DCs also have a relatively short lifespan (up to two weeks)^66^. These characteristics pose significant challenges for properly equipping DCs for the T- and B-cell priming processes. One common approach is to treat DCs with GM-CSF or M-CSF, which has long been used to differentiate DCs from bone marrow precursor cells and maintain the survival and proliferation of DCs. Indeed, Sipuleucel-T (Provenge), the first FDA-approved therapeutic cancer vaccine, utilizes the treatment of monocyte-derived DCs with a fusion protein of GM-CSF and prostatic acid phosphatase (PAP) antigen^67^. Upon infusion into patients, the engineered DCs are expected to exhibit improved survival and activation, an enhanced antigen-specific CTL response, and improved antitumor efficacy. Approaches based on various materials, including the use of nanoparticles, microparticles, polymer conjugates, and polymeric gels, have also been explored for the controlled exposure of immunomodulatory cytokines (e.g., GM-CSF) to DCs (Fig. 4a)^68^. Immature DCs can also be recruited to GM-CSF-releasing macroporous materials such as alginate hydrogels and mesoporous silica rods, where they can be modulated by cytokines and adjuvants in situ (Fig. 4b)^69,70^. DCs have been virally transduced with GM-CSF to enable stable expression. These transduced DCs can consistently express GM-CSF to drive self-proliferation and activation and show an improved ability to present tumor antigens and prime antigen-specific T and B cells^63,64^. In addition to GM-CSF, the overexpression of Fms-like tyrosine kinase 3 (Flt3) ligands in DCs via viral transduction methods has also been actively explored to enrich the population of type 1 conventional DCs (cDC1s), a subtype of DCs with superior antigen cross-presentation and priming of effector CD8^+^ T cells. Compared with the administration of unmodified DCs, the administration of Flt3 ligand-overexpressing DCs into tumor-bearing mice resulted in significantly greater numbers of cDC1s in the tumor microenvironment^28^.

### Overexpression of cytokines that affect neighboring T and B cells

**Fig. 5:**
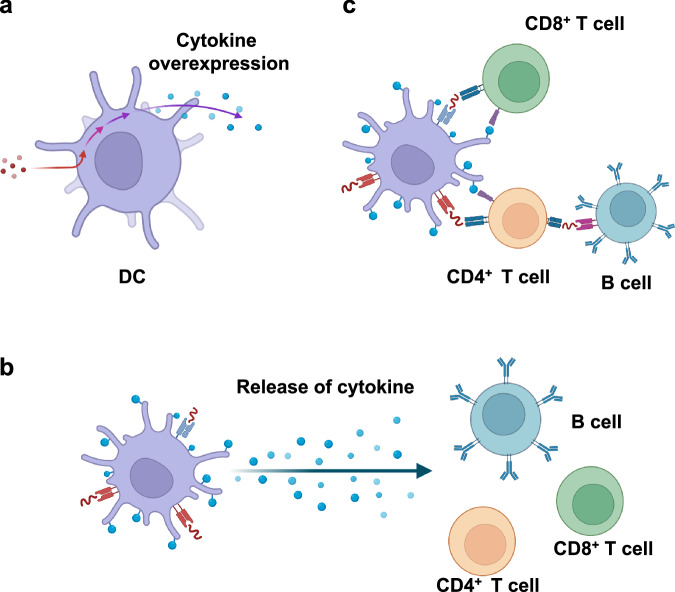
Overexpression of cytokines in and on DCs. **a** Cytokines can be overexpressed through the genetic engineering of DCs and subsequently released. (**b**) Cytokines overexpressed in DCs are released and affect neighboring T and B cells. **c** Overexpressed cytokines can be displayed on DC membranes to directly orchestrate the priming processes of T and B cells while avoiding the systemic release of cytokines.

In addition to overexpressing cytokines that can maintain the proliferation and activation of DCs, genetic engineering of DCs to overexpress proinflammatory cytokines that can drive the proliferation and maturation of T and B cells has also been actively explored (Fig. 5a). In these designs, cytokines are expressed in DCs and then are gradually released from DCs to modulate neighboring immune cells, such as T cells and B cells (Fig. 5b). For example, DCs transduced with IL-2 or IL-15 were shown to result in improved expansion of antigen-specific CD8^+^ T cells and to elicit a stronger CTL response and antitumor efficacy^71–73^. IL-12-transduced DCs were also shown to enrich CD8^+^ T cells in the tumor microenvironment compared with nontransduced DCs^74^. In addition to T helper 1 (Th1) cytokines (e.g., IL-2, IL-12, and IL-15), genetic engineering of DCs to overexpress T helper 2 (Th2) cytokines such as IL-4 and IL-10 has also been explored^75–77^. For example, transducing DCs with IL-4 could lead to augmented antibody responses by orchestrating the differentiation of CD4^+^ Th2 cells and B cells^78^. These approaches to utilizing DCs to produce and release cytokines locally could lead to enhanced CTL and humoral responses and improved therapeutic efficacy while reducing the off-target side effects of conventional cytokine therapies.

## Overexpression of cytokines on the DC membrane

While overexpressing cytokines inside DCs and utilizing engineered DCs to release produced cytokines have shown great promise for improving the overall CTL response with reduced side effects, issues regarding the systemic release of cytokines from engineered DCs remain^79^. Additionally, compared with the paracrine release of intracellular cytokines, the surface display of T/B-cell-effector cytokines could provide a unique approach to regulate the intercellular interactions between DCs and T/B cells (Fig. 5c)^80^. In view of these findings, efforts have been made to overexpress cytokines on the membrane of DCs. The substantial progress in the field of genetic engineering over the past few decades has provided powerful tools to overexpress different types and amounts of cytokines on DCs. The surface expression of cytokines (e.g., IL-2, IL-12, and IL-15) can be achieved by transducing DCs with a plasmid that encodes a fusion protein of the cytokine and a transmembrane domain^80^. The membrane-bound cytokines on DCs can directly modulate interacting T and B cells in a juxtacrine manner^81^. In this approach, DCs can simultaneously present MHC antigens, costimulatory signals, and cytokines to interacting immune cells, such as T and B cells, to drive improved priming of antigen-specific T and B cells. Compared with soluble cytokines in the surrounding milieu, membrane-displayed cytokines can stimulate interacting T and B cells in a timely manner and likely require a much lower concentration to achieve a comparable or better T/B-cell priming effect. For example, by transducing DCs with membrane-bound IL-12, engineered DCs significantly improved the stimulation of CD8^+^ T cells and the polarization of Th1 cells in the tumor microenvironment, resulting in a minimal concentration of IL-12 in the bloodstream^82^. Compared with soluble cytokines, membrane-bound IL-4 was also shown to stimulate IL-12 production in adjacent antigen-presenting cells and lead to enhanced antitumor efficacy^83^. These findings suggest that the overexpression of cytokines in membrane-bound forms represents a viable strategy to improve therapeutic efficacy while reducing off-target side effects.

## Surface conjugation of cytokines to DCs

**Fig. 6:**
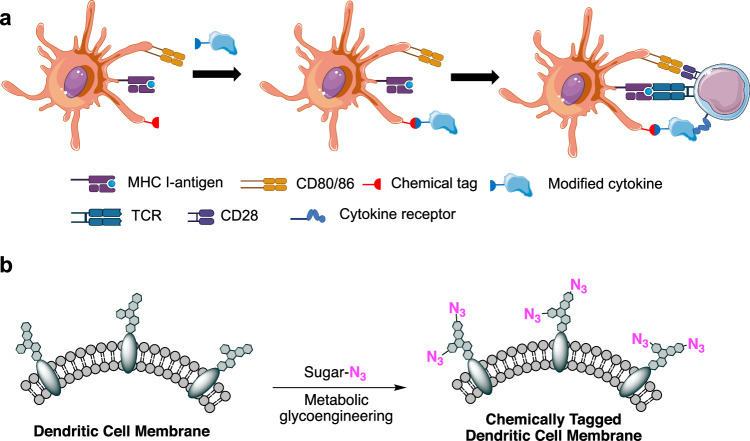
Cell surface modification methods for engineering DCs. **a** Schematic illustration of the conjugation of cytokines to surface-modified antigen-presenting DCs and the subsequent priming of T cells. **b** Metabolic labeling of DCs with azido groups via the metabolic glycoengineering process of unnatural sugars for the subsequent conjugation of cytokines via efficient click chemistry. Adapted from Han et al.^94^.

In addition to genetic engineering methods to overexpress cytokines on DC membranes, methods that involve the direct conjugation of cytokines onto DC membranes have also been explored (Fig. 6a). Compared with genetic engineering methods, these chemical approaches are simpler and avoid the safety concerns associated with viral transduction^84,85^. Conventional strategies to chemically attach cargos to the cell membrane either utilize hydrophobic or electrostatic interactions between cargos and the DC membrane or leverage amine‒carboxyl chemistry^86,87^. For example, lipid molecules with hydrophobic tails (e.g., dioleoyl, dipalmitoyl, or dimiristoyl lipids) can insert into the lipid bilayer structure of cell membranes. Cargos, via simple modification with N-hydroxysuccinimide (NHS) ester functional groups, can be directly conjugated to amine-bearing glycoproteins and glycolipids on the cell membrane^88–90^.

DCs can also be metabolically labeled with clickable chemical tags (e.g., azido groups) via the metabolic glycoengineering process of unnatural sugars for the subsequent conjugation of cytokines via efficient and bioorthogonal click chemistry^91,92^. Upon endocytosis by DCs, unnatural sugars bearing unique functional groups can undergo metabolic glycoengineering, conjugate to proteins and lipids, and become expressed on the cell membrane in the form of glycoproteins and glycolipids^93^. For example, DCs can be metabolically labeled with azido groups via treatment with tetraacetyl-*N*-azidoacetylmannosamine (Ac_4_ManAz) or poly(azido-sugar). The cell-surface azido groups can then capture dibenzocyclooctyne (DBCO)-modified cytokines (e.g., IL-2 and IL-15) via efficient click chemistry (Fig. 6b)^31,94^. This approach enables the targeted conjugation of cytokines to DCs in vitro and in vivo. The cytokines displayed on the surface of DCs can provide a continuous activation signal to interacting T cells for improved T-cell priming. Compared with nonconjugated DCs, IL-2- or IL-15-conjugated DCs resulted in an enhanced CTL response and antitumor efficacy in mouse models of lymphoma and melanoma^31,94^.

## Conclusion and Future Perspectives

Cytokines are crucial for the survival, proliferation, and differentiation of immune cells, as well as for regulating interactions among diverse immune cell types. However, despite the FDA approval of IFN-α and IL-2 for cancer treatment, conventional cytokine therapy is associated with low response rates and high toxicity. The use of DCs as the cytokine carrier via the forced overexpression of cytokines in DCs has emerged as a promising strategy to concentrate cytokines at the interface of DCs and T/B cells and thus amplify the generation and expansion of antigen-specific T and B cells. This approach can potentially reduce the doses of cytokines and associated side effects to achieve an enhanced CTL response and antitumor efficacy. Depending on the plasmid design, the overexpressed cytokines can either be released from DCs to exert a paracrine effect on interacting T and B cells or be displayed on DC membranes to modulate DC–T/B-cell interactions in a juxtacrine manner. At present, IL-2, IL-15, IL-12, IFN-α, and IL-4 are the most commonly studied cytokines, and the overexpression of various other types of cytokines in DCs could provide new opportunities for the modulation of T- and B-cell priming processes and the development of improved immunotherapies. Viral transduction is the most commonly used method for the forced expression of proteins in cells such as DCs because of the high transfection efficiency and availability of standard protocols. However, safety concerns related to viral transduction methods remain for potential clinical translation. The delivery of mRNAs could provide a safer and viable approach for the overexpression of cytokines in DCs in future efforts. Additionally, the substantial progress in mRNA chemistry over the past decades has enabled the design of mRNAs that encode cell-secreted proteins or surface-displayed proteins^95^.

In addition to the overexpression of cytokines in DCs via genetic engineering methods, recent advances in the chemical modification of cell membranes have opened new opportunities for the facile display of cytokines on DCs. The attachment of a hydrophobic lipid moiety to cytokines can improve their insertion into cell membranes via hydrophobic interactions, although the membrane retention time varies with the type of molecule. Endogenous functional groups (e.g., amino groups) from cell-surface proteins, lipids, or saccharides also enable the covalent conjugation of cytokines, but the conjugation efficiency is often limited by the low density of cell-surface reactive functional groups. Metabolic glycan labeling technology has provided a facile yet powerful approach for introducing clickable chemical tags (e.g., azido groups) onto the membrane of DCs for the subsequent conjugation of cytokines via efficient click chemistry. This method enables the display of tunable amounts of cytokines on the surface of DCs for the direct modulation of interacting T and B cells and has shown promise for improving the overall CTL response and antitumor efficacy against various types of cancer. Further endeavors will seek to further understand and optimize the stability, conformation, and membrane retention time of cytokines introduced onto DC membranes to more precisely orchestrate the interactions between DCs and T/B cells.

The choice of the type of cytokine for overexpression in DCs is inevitably critical and requires scrutiny for different scenarios of DC–T/B-cell interactions in the context of different cancers. The specific subtypes of T cells, such as CD8^+^ T cells, CD4^+^ effector T cells, and regulatory T cells, targeted by engineered DCs are key considerations. Equally important is the choice of releasing the overexpressed cytokines in a paracrine manner or displaying the cytokines on DC membranes, depending on the specific need of each scenario. At present, one cannot predict the ideal dose of cytokines that DCs should display to the interacting T and B cells for the optimal CTL response and antitumor efficacy. More efforts are needed to decipher the immune synapse between DCs and T/B cells and the mechanism underlying the cytokine-mediated modulation of those interactions. Nevertheless, the use of DCs as carriers of cytokines holds great promise for maximizing the impact of T/B-cell-effector cytokines while reducing the needed dose and associated toxicity compared with conventional cytokine therapies. A comprehensive investigation of how cytokines released or displayed by DCs dictate the phenotypes, activation status, and functions of T and B cells will also provide an improved understanding of the T- and B-cell priming processes and guide the design of new immunotherapies.

The DC vaccine, which involves ex vivo engineering of DCs with tumor antigens and cytokines, was among the first FDA-approved cancer immunotherapy ies (i.e., Sipuleucel-T). However, the modest therapeutic benefit (4.1-month improvement in the median survival of patients with prostate cancer) has hindered its wide use^96^. Over the past two decades, various other types of cancer vaccines, including tumor lysate vaccines, tumor exosome vaccines, nanovaccines, biomaterial scaffold vaccines, mRNA vaccines, and DNA vaccines, have been developed, but their antitumor efficacy is still far from satisfactory, and their clinical translation has been slow^97,98^. DC vaccines are still among the most promising cancer vaccine platforms for clinical translation, with one product already receiving FDA approval^99^. Indeed, extensive efforts have been made to improve the manufacturing and culture of DCs, optimize the subtypes of DCs, and enhance the in vivo persistence of DCs after adoptive transfer^100^. The overexpression of cytokines in DCs, as described in this review, represents a promising approach to increase the therapeutic benefits of DC vaccines and other DC-based immunotherapies.

## References

[1] Mellman, I., Coukos, G. & Dranoff, G. Cancer immunotherapy comes of age. *Nature* **480**, 480–489 (2011). 22193102 10.1038/nature10673 PMC3967235

[2] Blass, E. & Ott, P. A. Advances in the development of personalized neoantigen-based therapeutic cancer vaccines. *Nat. Rev. Clin. Oncol.* **18**, 215–229 (2021). 33473220 10.1038/s41571-020-00460-2 PMC7816749

[3] Wong, K. K., Li, W. A., Mooney, D. J. & Dranoff, G. Advances in therapeutic cancer vaccines. *Adv. Immunol.* **130**, 191–249 (2016). 26923002 10.1016/bs.ai.2015.12.001

[4] Chudnovskiy, A., Pasqual, G. & Victora, G. D. Studying interactions between dendritic cells and T cells in vivo. *Curr. Opin. Immunol.* **58**, 24–30 (2019). 30884422 10.1016/j.coi.2019.02.002 PMC6927575

[5] Bhatta, R. et al. Metabolic tagging of extracellular vesicles and development of enhanced extracellular vesicle based cancer vaccines. *Nat. Commun.* **14**, 8047 (2023). 38052869 10.1038/s41467-023-43914-8 PMC10697976

[6] Steinman, R. M. Lasker Basic Medical Research Award. Dendritic cells: versatile controllers of the immune system. *Nat. Med.* **13**, 1155–1159 (2007). 17917664 10.1038/nm1643

[7] Giza, H. M. & Bozzacco, L. Unboxing dendritic cells: tales of multi-faceted biology and function. *Immunology* **164**, 433–449 (2021). 34309853 10.1111/imm.13394 PMC8517577

[8] Bourque, J. & Hawiger, D. Immunomodulatory bonds of the partnership between dendritic cells and T Cells. *Crit. Rev. Immunol.* **38**, 379–401 (2018). 30792568 10.1615/CritRevImmunol.2018026790 PMC6380512

[9] LeBien, T. W. & Tedder, T. F. B lymphocytes: how they develop and function. *Blood* **112**, 1570–1580 (2008). 18725575 10.1182/blood-2008-02-078071 PMC2518873

[10] Kishimoto, T., Taga, T. & Akira, S. Cytokine signal transduction. *Cell* **76**, 253–262 (1994). 8293462 10.1016/0092-8674(94)90333-6

[11] Balkwill, F. R. & Burke, F. The cytokine network. *Immunol. Today* **10**, 299–304 (1989). 2686679 10.1016/0167-5699(89)90085-6

[12] Achar, S. R. et al. Universal antigen encoding of T cell activation from high-dimensional cytokine dynamics. *Science* **376**, 880–884 (2022). 35587980 10.1126/science.abl5311 PMC12302665

[13] Koneru, M., O’Cearbhaill, R., Pendharkar, S., Spriggs, D. R. & Brentjens, R. J. A phase I clinical trial of adoptive T cell therapy using IL-12 secreting MUC-16(ecto) directed chimeric antigen receptors for recurrent ovarian cancer. *J. Transl. Med.* **13**, 102 (2015). 25890361 10.1186/s12967-015-0460-x PMC4438636

[14] Waldmann, T. A. Cytokines in cancer immunotherapy. *Cold Spring Harb. Perspect Biol.* **10** (2018). 10.1101/cshperspect.a028472 PMC6280701 29101107

[15] Duggan, M. C. et al. A phase I study of recombinant (r) vaccinia-CEA(6D)-TRICOM and rFowlpox-CEA(6D)-TRICOM vaccines with GM-CSF and IFN-alpha-2b in patients with CEA-expressing carcinomas. *Cancer Immunol. Immunother.* **65**, 1353–1364 (2016). 27581603 10.1007/s00262-016-1893-7 PMC5071149

[16] Aggarwal, B. B., Gupta, S. C. & Kim, J. H. Historical perspectives on tumor necrosis factor and its superfamily: 25 years later, a golden journey. *Blood* **119**, 651–665 (2012). 22053109 10.1182/blood-2011-04-325225 PMC3265196

[17] Berraondo, P. et al. Cytokines in clinical cancer immunotherapy. *Br. J. Cancer* **120**, 6–15 (2019). 30413827 10.1038/s41416-018-0328-y PMC6325155

[18] Rochman, Y., Spolski, R. & Leonard, W. J. New insights into the regulation of T cells by gamma(c) family cytokines. *Nat. Rev. Immunol.* **9**, 480–490 (2009). 19543225 10.1038/nri2580 PMC2814538

[19] Gutterman, J. U. et al. Leukocyte interferon-induced tumor regression in human metastatic breast cancer, multiple myeloma, and malignant lymphoma. *Ann. Intern. Med.* **93**, 399–406 (1980). 6159812 10.7326/0003-4819-93-3-399

[20] Windbichler, G. H. et al. Interferon-gamma in the first-line therapy of ovarian cancer: a randomized phase III trial. *Br. J. Cancer* **82**, 1138–1144 (2000). 10735496 10.1054/bjoc.1999.1053 PMC2363351

[21] Jiang, T., Zhou, C. & Ren, S. Role of IL-2 in cancer immunotherapy. *Oncoimmunology* **5**, e1163462 (2016). 27471638 10.1080/2162402X.2016.1163462 PMC4938354

[22] Abrams, D. I. & Volberding, P. A. Alpha interferon therapy of AIDS-associated Kaposi’s sarcoma. *Semin Oncol.* **13**, 43–47 (1986). 3532335

[23] Conlon, K. C., Miljkovic, M. D. & Waldmann, T. A. Cytokines in the treatment of cancer. *J. Interferon Cytokine Res.* **39**, 6–21 (2019). 29889594 10.1089/jir.2018.0019 PMC6350412

[24] Maecker, H. T., Umetsu, D. T., DeKruyff, R. H. & Levy, S. DNA vaccination with cytokine fusion constructs biases the immune response to ovalbumin. *Vaccine* **15**, 1687–1696 (1997). 9364701 10.1016/s0264-410x(97)00088-1

[25] Auderset, F., Belnoue, E., Mastelic-Gavillet, B., Lambert, P. H. & Siegrist, C. A. A TLR7/8 agonist-including DOEPC-based cationic liposome formulation mediates its adjuvanticity through the sustained recruitment of highly activated monocytes in a Type I IFN-Independent but NF-kappaB-dependent manner. *Front Immunol.* **11**, 580974 (2020). 33262759 10.3389/fimmu.2020.580974 PMC7686571

[26] Sabado, R. L., Balan, S. & Bhardwaj, N. Dendritic cell-based immunotherapy. *Cell Res.* **27**, 74–95 (2017). 28025976 10.1038/cr.2016.157 PMC5223236

[27] Luchner, M., Reinke, S. & Milicic, A. TLR Agonists as vaccine adjuvants targeting cancer and infectious diseases. *Pharmaceutics* **13** (2021). 10.3390/pharmaceutics13020142 PMC7911620 33499143

[28] Ghasemi, A. et al. Cytokine-armed dendritic cell progenitors for antigen-agnostic cancer immunotherapy. *Nat. Cancer*10.1038/s43018-023-00668-y (2023). 37996514 10.1038/s43018-023-00668-y PMC10899110

[29] Pan, W. Y. et al. Cancer immunotherapy using a membrane-bound interleukin-12 with B7-1 transmembrane and cytoplasmic domains. *Mol. Ther.* **20**, 927–937 (2012). 22334018 10.1038/mt.2012.10 PMC3345985

[30] Chakrabarti, R., Chang, Y., Song, K. & Prud’homme, G. J. Plasmids encoding membrane-bound IL-4 or IL-12 strongly costimulate DNA vaccination against carcinoembryonic antigen (CEA). *Vaccine* **22**, 1199–1205 (2004). 15003648 10.1016/j.vaccine.2003.09.023

[31] Wang, H. et al. Metabolic labeling and targeted modulation of dendritic cells. *Nat. Mater.* **19**, 1244–1252 (2020). 32424368 10.1038/s41563-020-0680-1 PMC7748064

[32] Chen, D. S. & Mellman, I. Oncology meets immunology: the cancer-immunity cycle. *Immunity* **39**, 1–10 (2013). 23890059 10.1016/j.immuni.2013.07.012

[33] Quesada, J. R. et al. Treatment of hairy cell leukemia with recombinant alpha-interferon. *Blood* **68**, 493–497 (1986). 3730612

[34] Isaacs, A. & Lindenmann, J. The interferon. *Proc. R. Soc. Lond. B Biol. Sci.* **147**, 258–267 (1957). 26297790

[35] Jonasch, E. & Haluska, F. G. Interferon in oncological practice: review of interferon biology, clinical applications, and toxicities. *Oncologist* **6**, 34–55 (2001). 11161227 10.1634/theoncologist.6-1-34

[36] Rosenberg, S. A. IL-2: the first effective immunotherapy for human cancer. *J. Immunol.* **192**, 5451–5458 (2014). 24907378 10.4049/jimmunol.1490019 PMC6293462

[37] Boyman, O. & Sprent, J. The role of interleukin-2 during homeostasis and activation of the immune system. *Nat. Rev. Immunol.* **12**, 180–190 (2012). 22343569 10.1038/nri3156

[38] Andersen, R. et al. Tumor infiltrating lymphocyte therapy for ovarian cancer and renal cell carcinoma. *Hum. Vaccin Immunother.* **11**, 2790–2795 (2015). 26308285 10.1080/21645515.2015.1075106 PMC5054777

[39] Dutcher, J. P. et al. High dose interleukin-2 (Aldesleukin) - expert consensus on best management practices-2014. *J. Immunother. Cancer* **2**, 26 (2014). 31546315 10.1186/s40425-014-0026-0 PMC6889624

[40] Conlon, K. C. et al. Redistribution, hyperproliferation, activation of natural killer cells and CD8 T cells, and cytokine production during first-in-human clinical trial of recombinant human interleukin-15 in patients with cancer. *J. Clin. Oncol.* **33**, 74–82 (2015). 25403209 10.1200/JCO.2014.57.3329 PMC4268254

[41] Conlon, K. C. et al. IL15 by continuous intravenous infusion to adult patients with solid tumors in a phase I trial induced dramatic NK-cell subset expansion. *Clin. Cancer Res*. **25**, 4945–4954 (2019). 31142503 10.1158/1078-0432.CCR-18-3468 PMC6697593

[42] Sportes, C. et al. Phase I study of recombinant human interleukin-7 administration in subjects with refractory malignancy. *Clin. Cancer Res.* **16**, 727–735 (2010). 20068111 10.1158/1078-0432.CCR-09-1303 PMC2808195

[43] Leonard, J. P. et al. Effects of single-dose interleukin-12 exposure on interleukin-12-associated toxicity and interferon-gamma production. *Blood* **90**, 2541–2548 (1997). 9326219

[44] Steele, N. et al. A phase 1 trial of recombinant human IL-21 in combination with cetuximab in patients with metastatic colorectal cancer. *Br. J. Cancer* **106**, 793–798 (2012). 22315057 10.1038/bjc.2011.599 PMC3305963

[45] Zanna, M. Y. et al. Review of dendritic cells, their role in clinical immunology, and distribution in various animal species. *Int. J. Mol. Sci.* **22** (2021). 10.3390/ijms22158044 PMC8348663 34360810

[46] Nobs, S. P., Kayhan, M. & Kopf, M. GM-CSF intrinsically controls eosinophil accumulation in the setting of allergic airway inflammation. *J. Allergy Clin. Immunol.* **143**, 1513–1524.e1512 (2019). 30244025 10.1016/j.jaci.2018.08.044

[47] Hivroz, C., Chemin, K., Tourret, M. & Bohineust, A. Crosstalk between T lymphocytes and dendritic cells. *Crit. Rev. Immunol.* **32**, 139–155 (2012). 23216612 10.1615/critrevimmunol.v32.i2.30

[48] Rush, C. M. et al. Characterization of CD4+ T-cell-dendritic cell interactions during secondary antigen exposure in tolerance and priming. *Immunology* **128**, 463–471 (2009). 19930039 10.1111/j.1365-2567.2009.03124.x PMC2792131

[49] Cohen, M. et al. The interaction of CD4(+) helper T cells with dendritic cells shapes the tumor microenvironment and immune checkpoint blockade response. *Nat. Cancer* **3**, 303–317 (2022). 35241835 10.1038/s43018-022-00338-5

[50] Clark, E. A. Regulation of B lymphocytes by dendritic cells. *J. Exp. Med.* **185**, 801–803 (1997). 9120385 10.1084/jem.185.5.801 PMC2196172

[51] Lee, S. & Kim, T. D. Breakthroughs in cancer immunotherapy: an overview of T Cell, NK Cell, Mphi, and DC-based treatments. *Int. J. Mol. Sci.* **24** (2023). 10.3390/ijms242417634 PMC10744055 38139461

[52] Costa, S., Bevilacqua, D., Cassatella, M. A. & Scapini, P. Recent advances on the crosstalk between neutrophils and B or T lymphocytes. *Immunology* **156**, 23–32 (2019). 30259972 10.1111/imm.13005 PMC6283649

[53] Wang, G. et al. Crosstalk between dendritic cells and immune modulatory agents against sepsis. *Genes (Basel)* **11** (2020). 10.3390/genes11030323 PMC7140865 32197507

[54] Peng, X., He, Y., Huang, J., Tao, Y. & Liu, S. Metabolism of dendritic cells in tumor microenvironment: for immunotherapy. *Front Immunol.* **12**, 613492 (2021). 33732237 10.3389/fimmu.2021.613492 PMC7959811

[55] Gupta, Y. H., Khanom, A. & Acton, S. E. Control of dendritic cell function within the tumour microenvironment. *Front Immunol.* **13**, 733800 (2022). 35355992 10.3389/fimmu.2022.733800 PMC8960065

[56] Said, A. & Weindl, G. Regulation of dendritic cell function in inflammation. *J. Immunol. Res*. **2015**, 743169 (2015). 26229971 10.1155/2015/743169 PMC4503598

[57] Del Prete, A. et al. Dendritic cell subsets in cancer immunity and tumor antigen sensing. *Cell Mol. Immunol.* **20**, 432–447 (2023). 36949244 10.1038/s41423-023-00990-6 PMC10203372

[58] Tai, Y., Wang, Q., Korner, H., Zhang, L. & Wei, W. Molecular mechanisms of T cells activation by dendritic cells in autoimmune diseases. *Front Pharm.* **9**, 642 (2018). 10.3389/fphar.2018.00642 PMC6028573 29997500

[59] Alfei, F., Ho, P. C. & Lo, W. L. DCision-making in tumors governs T cell anti-tumor immunity. *Oncogene* **40**, 5253–5261 (2021). 34290401 10.1038/s41388-021-01946-8 PMC8390370

[60] Lee, K. M. C., Achuthan, A. A. & Hamilton, J. A. GM-CSF: a promising target in inflammation and autoimmunity. *Immunotargets Ther.* **9**, 225–240 (2020). 33150139 10.2147/ITT.S262566 PMC7605919

[61] Yang, Y. & Lundqvist, A. Immunomodulatory effects of IL-2 and IL-15; implications for cancer immunotherapy. *Cancers (Basel)* **12** (2020). 10.3390/cancers12123586 PMC7761238 33266177

[62] Makker, S., Galley, C. & Bennett, C. L. Cancer vaccines: from an immunology perspective. *Immunother. Adv.* **4**, ltad030 (2024). 38223410 10.1093/immadv/ltad030 PMC10787373

[63] Tenbusch, M. et al. Coexpression of GM-CSF and antigen in DNA prime-adenoviral vector boost immunization enhances polyfunctional CD8+ T cell responses, whereas expression of GM-CSF antigen fusion protein induces autoimmunity. *BMC Immunol.* **9**, 13 (2008). 18405363 10.1186/1471-2172-9-13 PMC2324072

[64] Stam, A. G. et al. CD40-targeted adenoviral GM-CSF gene transfer enhances and prolongs the maturation of human CML-derived dendritic cells upon cytokine deprivation. *Br. J. Cancer* **89**, 1162–1165 (2003). 14520439 10.1038/sj.bjc.6601225 PMC2394320

[65] Jones, D. S. et al. Cell surface-tethered IL-12 repolarizes the tumor immune microenvironment to enhance the efficacy of adoptive T cell therapy. *Sci. Adv.* **8**, eabi8075 (2022). 35476449 10.1126/sciadv.abi8075 PMC9045725

[66] Nayar, S., Dasgupta, P. & Galustian, C. Extending the lifespan and efficacies of immune cells used in adoptive transfer for cancer immunotherapies-A review. *Oncoimmunology* **4**, e1002720 (2015). 26155387 10.1080/2162402X.2014.1002720 PMC4489929

[67] Handy, C. E. & Antonarakis, E. S. Sipuleucel-T for the treatment of prostate cancer: novel insights and future directions. *Future Oncol.* **14**, 907–917 (2018). 29260582 10.2217/fon-2017-0531 PMC5925432

[68] Pires, I. S., Hammond, P. T. & Irvine, D. J. Engineering strategies for immunomodulatory cytokine therapies - challenges and clinical progress. *Adv. Ther. (Weinh)* **4** (2021). 10.1002/adtp.202100035 PMC8562465 34734110

[69] Super, M. et al. Biomaterial vaccines capturing pathogen-associated molecular patterns protect against bacterial infections and septic shock. *Nat. Biomed. Eng.* **6**, 8–18 (2022). 34239117 10.1038/s41551-021-00756-3

[70] Wang, H. Immune cell homing biomaterials for immunotherapy. *Acc. Mater. Res.* **1**, 172–174 (2020).

[71] Van den Bergh, J. et al. Transpresentation of interleukin-15 by IL-15/IL-15Ralpha mRNA-engineered human dendritic cells boosts antitumoral natural killer cell activity. *Oncotarget* **6**, 44123–44133 (2015). 26675759 10.18632/oncotarget.6536 PMC4792546

[72] Tourkova, I. L. et al. Increased function and survival of IL-15-transduced human dendritic cells are mediated by up-regulation of IL-15Ralpha and Bcl-2. *J. Leukoc. Biol.* **72**, 1037–1045 (2002). 12429727

[73] Yang, J. Y., Li, X., Gao, L., Teng, Z. H. & Liu, W. C. Co-transfection of dendritic cells with AFP and IL-2 genes enhances the induction of tumor antigen-specific antitumor immunity. *Exp. Ther. Med.* **4**, 655–660 (2012). 23170121 10.3892/etm.2012.635 PMC3501441

[74] Mierzejewska, J. et al. The beneficial effect of IL-12 and IL-18 transduced dendritic cells stimulated with tumor antigens on generation of an antitumor response in a mouse colon carcinoma model. *J. Immunol. Res*. **2022**, 7508928 (2022). 35372586 10.1155/2022/7508928 PMC8975686

[75] Scheerlinck, J. P. et al. The immune response to a DNA vaccine can be modulated by co-delivery of cytokine genes using a DNA prime-protein boost strategy. *Vaccine* **19**, 4053–4060 (2001). 11427282 10.1016/s0264-410x(01)00133-5

[76] Ko, K. S., Lee, M., Koh, J. J. & Kim, S. W. Combined administration of plasmids encoding IL-4 and IL-10 prevents the development of autoimmune diabetes in nonobese diabetic mice. *Mol. Ther.* **4**, 313–316 (2001). 11592833 10.1006/mthe.2001.0459

[77] Yen, H. H. & Scheerlinck, J. P. Co-delivery of plasmid-encoded cytokines modulates the immune response to a DNA vaccine delivered by in vivo electroporation. *Vaccine* **25**, 2575–2582 (2007). 17224210 10.1016/j.vaccine.2006.12.025

[78] Junttila, I. S. Tuning the cytokine responses: an update on Interleukin (IL)-4 and IL-13 Receptor Complexes. *Front Immunol.* **9**, 888 (2018). 29930549 10.3389/fimmu.2018.00888 PMC6001902

[79] Zhang, L. et al. Tumor-infiltrating lymphocytes genetically engineered with an inducible gene encoding interleukin-12 for the immunotherapy of metastatic melanoma. *Clin. Cancer Res* **21**, 2278–2288 (2015). 25695689 10.1158/1078-0432.CCR-14-2085 PMC4433819

[80] Nguyen, K. G. et al. Localized Interleukin-12 for Cancer Immunotherapy. *Front Immunol.* **11**, 575597 (2020). 33178203 10.3389/fimmu.2020.575597 PMC7593768

[81] Kim, Y. S. Tumor therapy applying membrane-bound form of cytokines. *Immune Netw.* **9**, 158–168 (2009). 20157604 10.4110/in.2009.9.5.158 PMC2816950

[82] D’Andrea, A., Ma, X., Aste-Amezaga, M., Paganin, C. & Trinchieri, G. Stimulatory and inhibitory effects of interleukin (IL)-4 and IL-13 on the production of cytokines by human peripheral blood mononuclear cells: priming for IL-12 and tumor necrosis factor alpha production. *J. Exp. Med.* **181**, 537–546 (1995). 7836910 10.1084/jem.181.2.537 PMC2191875

[83] Hochrein, H. et al. Interleukin (IL)-4 is a major regulatory cytokine governing bioactive IL-12 production by mouse and human dendritic cells. *J. Exp. Med.* **192**, 823–833 (2000). 10993913 10.1084/jem.192.6.823 PMC2193283

[84] Roy, S., Cha, J. N. & Goodwin, A. P. Nongenetic bioconjugation strategies for modifying cell membranes and membrane proteins: a review. *Bioconjug. Chem.* **31**, 2465–2475 (2020). 33146010 10.1021/acs.bioconjchem.0c00529 PMC7978361

[85] Custodio, C. A. & Mano, J. F. Cell surface engineering to control cellular interactions. *ChemNanoMat.* **2**, 376–384 (2016). 30842920 10.1002/cnma.201600047 PMC6398572

[86] Kato, K., Itoh, C., Yasukouchi, T. & Nagamune, T. Rapid protein anchoring into the membranes of Mammalian cells using oleyl chain and poly(ethylene glycol) derivatives. *Biotechnol. Prog.* **20**, 897–904 (2004). 15176897 10.1021/bp0342093

[87] Xie, R., Hong, S., Feng, L., Rong, J. & Chen, X. Cell-selective metabolic glycan labeling based on ligand-targeted liposomes. *J. Am. Chem. Soc.* **134**, 9914–9917 (2012). 22646989 10.1021/ja303853y

[88] Lim, K. S., Lee, D. Y., Valencia, G. M., Won, Y. W. & Bull, D. A. Cell surface-engineering to embed targeting ligands or tracking agents on the cell membrane. *Biochem Biophys. Res. Commun.* **482**, 1042–1047 (2017). 27908724 10.1016/j.bbrc.2016.11.155

[89] Contreras, J. L. et al. A novel approach to xenotransplantation combining surface engineering and genetic modification of isolated adult porcine islets. *Surgery* **136**, 537–547 (2004). 15349100 10.1016/j.surg.2004.05.031

[90] Yamamoto, T., Teramura, Y., Itagaki, T., Arima, Y. & Iwata, H. Interaction of poly(ethylene glycol)-conjugated phospholipids with supported lipid membranes and their influence on protein adsorption. *Sci. Technol. Adv. Mater.* **17**, 677–684 (2016). 27877914 10.1080/14686996.2016.1240006 PMC5101893

[91] Wang, H. & Mooney, D. J. Metabolic glycan labelling for cancer-targeted therapy. *Nat. Chem.* **12**, 1102–1114 (2020). 33219365 10.1038/s41557-020-00587-w

[92] Bhatta, R., Han, J., Zhou, J., Li, H. & Wang, H. Recyclable cell-surface chemical tags for repetitive cancer targeting. *J. Control Release* **347**, 164–174 (2022). 35537537 10.1016/j.jconrel.2022.05.007

[93] Chang, P. V. et al. Copper-free click chemistry in living animals. *Proc. Natl. Acad. Sci. USA* **107**, 1821–1826 (2010). 20080615 10.1073/pnas.0911116107 PMC2836626

[94] Han, J. et al. Metabolic glycan labeling immobilizes dendritic cell membrane and enhances antitumor efficacy of dendritic cell vaccine. *Nat. Commun.* **14**, 5049 (2023). 37598185 10.1038/s41467-023-40886-7 PMC10439884

[95] Dorrie, J., Schaft, N., Schuler, G. & Schuler-Thurner, B. Therapeutic cancer vaccination with ex vivo RNA-transfected dendritic cells-an update. *Pharmaceutics* **12** (2020). 10.3390/pharmaceutics12020092 PMC7076681 31979205

[96] Kantoff, P. W. et al. Sipuleucel-T immunotherapy for castration-resistant prostate cancer. *N. Engl. J. Med.* **363**, 411–422 (2010). 20818862 10.1056/NEJMoa1001294

[97] Melief, C. J., van Hall, T., Arens, R., Ossendorp, F. & van der Burg, S. H. Therapeutic cancer vaccines. *J. Clin. Invest.* **125**, 3401–3412 (2015). 26214521 10.1172/JCI80009 PMC4588240

[98] Wang, H. & Mooney, D. J. Biomaterial-assisted targeted modulation of immune cells in cancer treatment. *Nat. Mater.* **17**, 761–772 (2018). 30104668 10.1038/s41563-018-0147-9

[99] Palucka, K. & Banchereau, J. Dendritic-cell-based therapeutic cancer vaccines. *Immunity* **39**, 38–48 (2013). 23890062 10.1016/j.immuni.2013.07.004 PMC3788678

[100] Saxena, M. & Bhardwaj, N. Re-emergence of dendritic cell vaccines for cancer treatment. *Trends Cancer* **4**, 119–137 (2018). 29458962 10.1016/j.trecan.2017.12.007 PMC5823288

